# Natural compounds regulating fatty acid oxidation in the treatment of diabetic kidney disease

**DOI:** 10.3389/fnut.2025.1669557

**Published:** 2025-10-15

**Authors:** Jianing Sun, Chengqian Yin, Zhe Li, Xiangyu Gao, Songlin Li, Hua Gao, Yan An, Peng Liu, Na Liu

**Affiliations:** ^1^Renal Division, Department of Medicine, Heilongjiang Academy of Chinese Medicine Sciences, Harbin, China; ^2^Xiyuan Hospital, China Academy of Chinese Medical Sciences, Beijing, China

**Keywords:** diabetic kidney disease, renal lipid metabolism disorders, natural compounds, fatty acid oxidation, PPARs

## Abstract

Diabetic kidney disease (DKD) is one of the leading causes of end-stage renal disease worldwide, and lipid metabolism disorder is a key factor in accelerating its progression. Among them, the impaired fatty acid oxidation (FAO) function of renal tissue constitutes one of the core pathological links of lipid metabolism disorders. In DKD, impaired FAO function can directly lead to lipid accumulation, mitochondrial stress, and trigger an inflammatory cascade, thereby promoting the occurrence and development of glomerulosclerosis and renal tubular injury. However, the efficacy of current DKD treatment strategies is still limited. Natural compounds (such as polyphenols, phenolic acids, alkaloids, glycosides, and carotenoids) have shown potential in renal protection due to their multi-target and multi-pathway characteristics. Studies have shown that regulating the FAO process in the context of lipid metabolism disorders is a crucial mechanism by which natural compounds can exert anti-DKD effects. It is worth noting that peroxisome proliferator-activated receptors (PPARs) are core transcription factors that regulate FAO. Specifically, these active ingredients can upregulate the expression of their downstream target genes by activating the PPAR signaling pathway (especially PPARα), thereby improving FAO function, correcting abnormal lipid metabolism, and ultimately delaying the progression of renal pathological mechanisms such as inflammation and fibrosis. The above findings provide an essential scientific basis for the development of new, safe, and effective DKD therapeutic drugs.

## 1 Introduction

Diabetic Kidney Disease (DKD), one of the most common microvascular complications of diabetes, affects approximately 30–40% of diabetic patients who will ultimately develop the condition during the later stages of their disease (1, 2). DKD is characterized by persistent proteinuria and progressive renal dysfunction, with its pathological changes including renal enlargement, thickening of the basement membrane in the glomeruli, cell damage in the podocytes, as well as destruction of glomerular and tubular structures. Ultimately, it can progress to end-stage renal disease (3, 4). The pathogenesis of DKD involves multiple factors, such as the activation of inflammatory responses, imbalance of oxidative stress, and dysregulation of lipid metabolism, among which the nephrotoxicity caused by lipid accumulation has been confirmed as a key driving factor accelerating renal fibrosis (5–7). At present, the treatment of DKD focuses on comprehensive management strategies, including blood glucose control, blood pressure management, blood lipid regulation, and diet and lifestyle interventions (8). As of now, the drugs approved in the FDA database for delaying the progression of DKD mainly focus on RAAS inhibitors, SGLT2 inhibitors, and non-steroidal MRAs. They can improve glomerular permeability and enhance insulin sensitivity, with apparent renal and cardiovascular protective effects, and have become a standard component in the treatment of DKD (9). Among them, dapagliflozin and empagliflozin have their indications expanded to the CKD population (10). Finerenone, as the first approved non-steroidal MRA, has new capabilities in anti-inflammation and anti-fibrosis (11). Although these drugs have been proven to play a stable role in the treatment of DKD and are widely used in clinical practice, these treatments still have certain limitations (12).

Lipid metabolism disorder is not only an important driving factor for systemic diseases such as hypercholesterolemia, hypertriglyceridemia, diabetes, and atherosclerosis, but also plays a key role in the occurrence and development of DKD (13). The progression of DKD is characterized by significant lipid disorders, which are characterized by abnormal accumulation of lipids such as free fatty acids, diacylglycerols, and ceramides in kidney tissue, thereby triggering lipotoxicity. Hypoxia in the renal microenvironment inhibits fatty acid oxidation and promotes lipid synthesis through the HIF-1α pathway, while chronic inflammation further aggravates lipid metabolism disorders through inflammatory cytokines, inflammasomes and macrophage polarization (14). In addition, lipid metabolism may also involve mechanisms such as cell ferroptosis, lipid metabolism reprogramming, and immune regulation of intestinal microbiota (15). The damage to renal tubular epithelial cells (TECs) is the core link in the progression of DKD. Its intracellular lipid accumulation can inhibit mitochondrial fatty acid oxidation (FAO), resulting in reduced ATP synthesis and cell energy failure, thereby accelerating the deterioration of renal function. In podocytes, lipid deposition can directly induce cytoskeletal rearrangement, shedding, and apoptosis, thereby accelerating the process of fibrosis (16). The process of FAO is crucial in lipid metabolism, providing energy for cell function and participating in the inflammatory response of cells (17). In addition, the role of FAO in DKD has been confirmed by research (18, 19). Mitochondrial FAO is the main pathway of fatty acid (FA) degradation and is essential for maintaining human energy homeostasis. At the same time, FAO is the main energy source for the heart, skeletal muscle, and kidney. Inhibition of FAO can destroy the balance between the synthesis, uptake and consumption of fatty acids (FA) in renal cells through transcriptional regulation (such as STAT6-PPARα axis), destruction of renal microenvironment homeostasis (hypoxia, inflammation), and triggering mitochondrial dysfunction, eventually leading to abnormal lipid accumulation and renal injury (20, 21). Peroxisome proliferator-activated receptors (PPARs) belong to the ligand-activated nuclear receptor family and are key regulators of FAO. They can upregulate the expression of FAO-related genes such as carnitine palmitoyl transferase I (CPT1) and medium-chain acyl-CoA dehydrogenase (MCAD). PPARγ dominates lipid storage and homeostasis, while PPARα promotes fatty acid decomposition (22). Recent studies have found that renal lipid metabolism disorders in diabetic patients can aggravate insulin resistance and glomerulosclerosis. PPARα agonists can reduce lipid accumulation by activating the FAO pathway, thereby improving renal injury (23). Intervention with PPARα agonists, such as fenofibrate and pemafibrate, has been shown to restore the expression levels of FAO-related genes [e.g., carnitine palmitoyltransferase 1A (CPT1A), acyl-CoA oxidase 1 (ACOX1)] in animal models, while simultaneously improving mitochondrial homeostasis and alleviating renal injury (24, 25). On the other hand, although PPARγ agonists like pioglitazone exert reno-protective effects by improving mitochondrial function, reducing oxidative stress levels, and alleviating renal tubulointerstitial fibrosis, direct evidence regarding their ability to directly restore FAO function in renal tubular cells remains relatively limited (26). Notably, activation of both PPARα and PPARγ pathways via PPARα/γ dual agonists (e.g., tesaglitazar) or the combination of fenofibrate and pioglitazone can exhibit synergistically enhanced reno-protective effects, specifically including reducing proteinuria excretion, inhibiting renal inflammatory responses, and delaying the progression of fibrosis (27, 28). In summary, these findings clearly highlight the value of PPARα and PPARγ as key therapeutic targets for regulating renal lipid metabolism disorders in DKD. Targeting PPAR subtypes or combined regulation is expected to be a potential strategy to enhance DKD metabolic damage and pathological progression.

**Figure 1:**
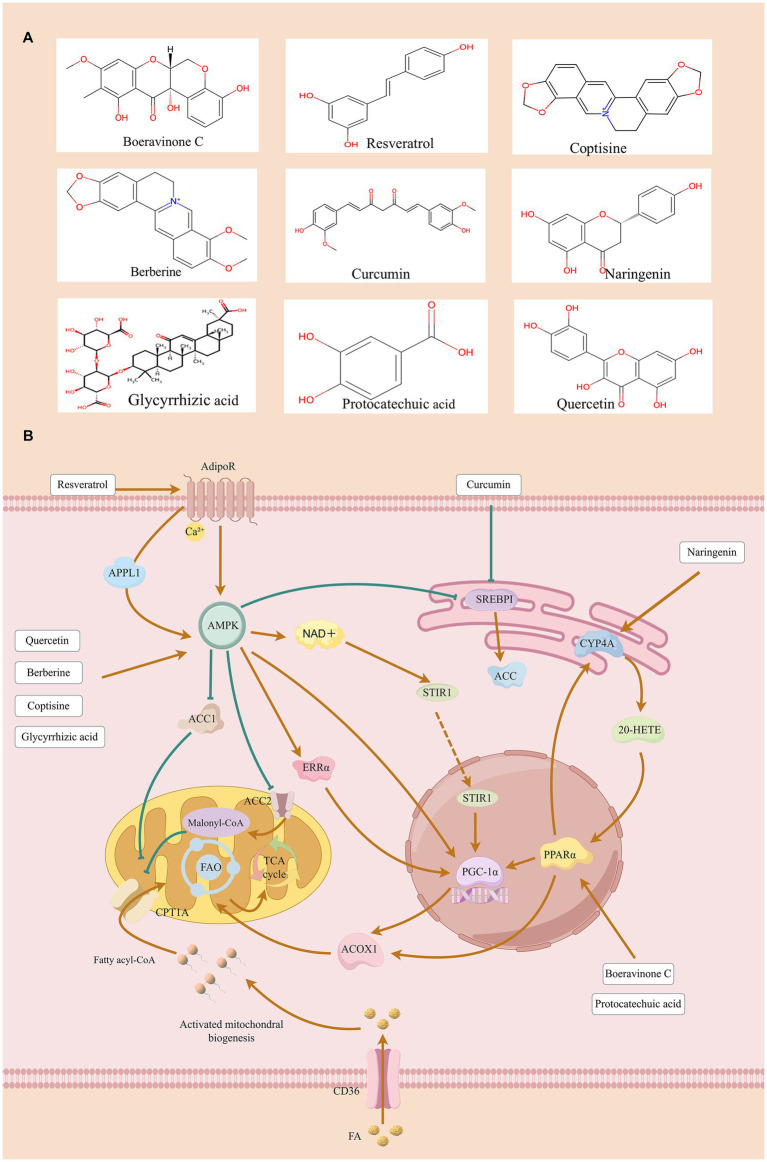
The protective effect of natural compounds on DKD by improving FAO.

Natural compounds have structurally clear chemical formulas, play a crucial role in drug discovery, and often exhibit multi-target synergistic effects in disease treatment (29). Natural compounds account for a significant portion of drugs approved by the FDA. For instance, in the field of oncology, half of the small-molecule drugs approved over the past 60 years are either natural compounds or directly derived from natural products (30). Modern pharmacological research and clinical trials have initially confirmed the potential of natural compounds in the treatment of DKD (31, 32). These active ingredients have a variety of pharmacological activities such as anti-inflammatory, anti-oxidative stress, anti-fibrosis, and regulation of lipid metabolism. They can inhibit pathological processes such as ferroptosis and autophagy, thereby improving the pathological state of DKD (33–35). It is worth noting that natural components can also correct lipid metabolism disorders in DKD by specifically regulating the activity of FAO key enzymes and related upstream and downstream signaling pathways (36). Therefore, this article aims to summarize the molecular mechanism of natural active ingredients improving DKD by targeting the FAO process to promote the discovery of DKD treatment. The relevant core mechanisms are summarized in Table 1 and shown in Figure 1. We believe that although there are deficiencies in research design and clinical trials, naturally active ingredients have a good prospect in the clinical transformation of DKD treatment.

**Table 1:** Natural compounds improve DKD through FAO.

Compounds	Compound components	Model	Mechanisms related to lipid metabolism	Signal passage	References
Polyphenols	Resveratrol	db/db mice; NMS2 mesangial cells	Improve lipid deposition; Inhibiting oxidative damage	AMPK/SIRT1/ PGC1α axis	(45)
		db/db mice; glomerular endothelial cell	Reduce lipid deposition; Inhibiting oxidative damage	AMPK/SIRT1/PGC-1α axis	(46)
	Boeravinone C	STZ mice; HK-2 cell	Improve lipid metabolism	PPARα pathway	(78)
	Quercetin	db/db mice	Regulation of fatty acid oxidation, Regulating oxidative stress	PPARα/FAO axis	(63)
	Naringenin	HFD mice; NRK-52E cell	Lipid metabolism regulation, Regulating oxidative stress	20-HETE/PPARs axis	(75)
	Curcumin	db/db mice; human podocytes	Improve lipid metabolism Reduce podocyte injury; Inhibiting oxidative damage	CXCL16/PPARγ pathway	(53)
Phenolic acids	Protocatechuic acid	STZ induced-mice	Regulating glucose homeostasis; Regulation of fatty acid oxidation	PPARα/γ/RAGE axis	(85)
Alkaloid	Berberine	db/db mice; HK-2 cells	Regulation of fatty acid oxidation; Improve mitochondrial function and biosynthesis	AMPK/PGC-1α pathway	(98)
	Coptisine	HK-2 cells	Reducing lipid droplet formation; Regulating the balance between lipid synthesis and FAO	AMPK/ACC/CPT-1 pathway	(103)
Glycoside	Glycyrrhizic acid	db/db mice mouse; mesangial cells (MMCs)	Reducing lipid accumulation and lipotoxicity; Promoting FAO	AMPK/SIRT1/PGC-1α pathway	(108)

## 2 Natural compounds regulating FAO to improve DKD

In this review, we searched PubMed for articles published from inception to June 29, 2025. Literature search was performed according to the following search terms (DKD or DN or Diabetic nephropathy or Diabetic kidney disease) and (singular or herbs or Natural ingredients or Natural produce or Natural compounds) and (PPARs or PPAR or FAO or Oxidation of fatty acid). Our inclusion criteria mainly include: Cell or animal experimental studies in the DKD model; Intervention treatment using natural active ingredients; and only retrieve English literature. The time range is from the establishment of the PubMed database to June 2025; Only experimental studies are included, excluding reviews and commentaries. A total of 10 studies were eligible, involving 9 different natural active ingredients. Use ClinicalTrials.gov, International Clinical Trials Registry Platform (ICTRP), China Clinical Trial Registry (ChiCTR), and European Union Clinical Trials Register (EUCTR) to monitor the clinical trials involved. We summarized the natural compounds used to treat DKD by regulating FAO, and divided them into polyphenols, phenolic acids, alkaloids, glycosides, and carotenoids according to their structural characteristics.

### 2.1 Polyphenols

Polyphenols are a class of natural compounds that are widely present in food. They are characterized by the presence of at least two phenolic groups associated with more or less complex structures, usually with high molecular weight (37). Research shows that polyphenols can exert anti-inflammatory, antioxidant, and lipid metabolism-regulating effects by inhibiting the JNK and NF-κB inflammatory signaling pathways, as well as activating the AKT/AS160/GLUT4 pathway, thereby improving metabolic function disorders (38). Epidemiological studies indicate that consuming high levels of natural polyphenols in the diet is linked to a decreased risk of various chronic illnesses, such as cardiovascular disease, type 2 diabetes, and chronic kidney disease (39). Notably, recent studies have demonstrated that polyphenols can regulate PPARα to influence energy storage, adipogenesis, upregulation of fatty acid metabolism, and β-oxidation processes in tissues. They can also engage PPARβ/*δ* to participate in the uptake and β-oxidation of fatty acids (FA) in muscle and white adipose tissue. Additionally, PPARγ mediates energy storage-adipogenesis, glucose metabolism, and inflammatory responses in white adipose tissue (WAT) and macrophages. The synergistic effects of the aforementioned PPAR subtypes collectively ameliorate the state of renal lipid metabolism disorders in patients with DKD (40).

Resveratrol (RV) is a common polyphenolic compound found in various plants such as grapes and berries, playing an essential role in the treatment of kidney diseases. Mechanism studies have shown that RV inhibits the SphK1-NF-κB inflammatory cascade and enhances LC3-II/Beclin1-mediated autophagic flux by activating the SIRT1 signaling pathway, thereby exerting a renal protective effect (41). At the level of lipid metabolism, RV can regulate the process of fat accumulation, promote fatty acid oxidation and lipolysis, and inhibit a variety of renal injury and fibrosis processes (42). However, resveratrol exhibits characteristics common to most natural active ingredients in terms of pharmacokinetics, namely low bioavailability and rapid metabolism (43). A double-blind trial of patients with type 2 diabetes and coronary heart disease confirmed that RV can upregulate the expression levels of PPARγ and SIRT1 in peripheral blood mononuclear cells (PBMCs) (44). In the DKD model, RV activates the AMPK/SIRT1/PPARα signaling axis by activating adiponectin receptors (AdipoR1/AdipoR2), induces AMPK phosphorylation, and enhances SIRT1/PGC-1α signaling, thereby regulating the downstream effector molecule PPARα/ERR-1α/SREBP1. Finally, RV protects the kidney by improving oxidative stress, inhibiting apoptosis, and increasing circulating adiponectin levels (45, 46). Although the vast majority of animal studies support that resveratrol can ameliorate renal pathology in DKD mice, conflicting results exist regarding its effects on key signaling pathways (AMPK/SIRT1). In dose-related studies, the effect of resveratrol exhibits a U-shaped curve, which supports the need for more refined dose–response clinical studies in the future (47).

Turmeric is a kind of Curcuma plant that is widely planted in Southeast Asia. The main active ingredient is curcumin (1,7-bis(4-hydroxy-3-methoxyphenyl)-1,6-heptadiene-3,5-dione) (48). As a lipophilic polyphenol classified as curcuminoids, it has anti-inflammatory and antioxidant effects (49). Recent studies have shown that curcumin can alleviate inflammation in chronic kidney disease (CKD) by reducing chemokine 2 (CCL-2), interferon-γ (IFN-γ), and interleukin-4 (IL-4) and regulating lipid peroxidation (50). Preclinical data from animal models and Phase I clinical studies conducted on human volunteers and cancer patients indicate that the systemic bioavailability of orally administered curcumin is low and exhibits rapid metabolic rates (51). A meta-analysis related to curcumin showed that curcumin treatment can significantly reduce serum creatinine (Scr), total cholesterol (TC), and fasting blood glucose (FBG) in patients with DKD, demonstrating potential efficacy (52). But it also found no statistically significant impact on indicators such as BUN and proteinuria, suggesting that its efficacy may be limited to specific pathological processes (52). Curcumin treatment of the db/db mice model can effectively reduce chemokine ligand CXCL16, sterol regulatory element binding protein 1 (SREBP1), SREBP2, and adipose differentiation-related protein (ADRP), and upregulate the expression of PPARγ in renal cortex. Studies have shown that curcumin can improve lipid metabolism disorders in podocytes and reduce glomerular lipid deposition through the CXCL16-PPARγ signaling pathway, thereby reducing the pathological damage of DKD (53).

Quercetin [Ka(3,3′,4′,5,7-Pentahydroxyflavone)] is a representative substance of flavonols (54). It is widely found in many plants and is known for its significant antioxidant, anti-inflammatory, and anti-diabetic properties (55). Studies have shown that quercetin can activate adipose tissue signal transduction, promote fatty acid β-oxidation, and reduce lipid deposition by up-regulating the expression of PPARα (56). It also down-regulated the expression of PPARγ and inhibited the activity of inflammatory factors mediated by the MAPK signaling pathway (57). Quercetin exhibits low bioavailability and extensive first-pass metabolism in pharmacokinetics. Animal studies have shown that after intravenous injection of quercetin in rats, it is widely distributed in the kidneys, liver, heart, and brain tissues (58). Only one clinical trial has shown that quercetin can reduce blood pressure in patients with stage 1 hypertension (59). We have noted that there are inconsistencies in the conclusions of existing literature regarding the mechanism of quercetin in relation to the PPAR signaling pathway. An early biochemical study demonstrated that quercetin and related lipoxygenase inhibitors can bind to the ligand-binding domain of PPAR, thereby inhibiting PPAR-mediated transcriptional processes in primary keratinocytes. This finding suggests that PPAR may be directly antagonized under certain conditions (60). In contrast, multiple subsequent studies on metabolic tissues and disease models have shown that quercetin can activate the LKB1-AMPK-SIRT1 signaling axis, thereby promoting FAO and upregulating the expression of PPARα-regulated targets (e.g., CPT1) (56, 61, 62). Furthermore, a study using a DKD model reported that the combination of Dasatinib and quercetin (DQ combination) can improve renal FAO function. Subsequent mechanistic studies, via molecular docking and genetic manipulation, have confirmed that the DQ combination promotes the FAO process by specifically upregulating the expression of key FAO enzymes CPT1A and ACOX1—through targeted activation of PPARα. Meanwhile, this combination also significantly reduces the expression of myofibroblast activation marker (α-SMA) and extracellular matrix proteins, providing experimental evidence for the involvement of the DQ combination in the restoration of PPARα-dependent FAO function in renal tissues (63). The above findings suggest that the effect of quercetin on PPARs appears to be dependent on the specific cellular or tissue context: in certain non-metabolic cells, it exhibits an inhibitory effect on PPARs, whereas in renal tissues, it promotes FAO by activating AMPK/PPARα-related pathways.

Naringenin (2, 3-dihydro-5,7-dihydroxy-2-(4-hydroxyphenyl)-4H-1-benzopyran-4-one) is a flavonoid compound widely found in the peel of citrus fruits (64). Its core biological functions include alleviating oxidative stress, improving lipid metabolism disorders, and thus exerting renal protective effects (65). Existing studies have shown that the compound can significantly improve the disease phenotype of mice models of hyperuricemia and acute kidney injury by reducing serum uric acid levels and alleviating pathological changes of renal fibrosis (66, 67). Molecular mechanism studies further revealed that naringenin inhibited the expression of SREBP-1c through activation of the AMPK signaling pathway, thereby reducing adipogenesis, while up-regulating the expression of PPARα and promoting fatty acid oxidation, thereby improving metabolic disorders and renal lipid metabolism (68–70). Research has found that the bioavailability of naringenin in rat and dog models is 44.1 and 34.4% respectively, indicating a significant first-pass effect; while in humans, it shows a longer half-life (approximately 48 h) and a notable gender difference in single-dose administration (71). Currently, the 10 registered clinical trials are insufficient to provide clear conclusions, mainly limited by the lack of pharmacokinetic data on naringenin, poor chemical stability, and complex metabolism (72). In studies related to diabetic models, the therapeutic effects of naringenin have shown contradictions, with conflicting conclusions reported across different studies. On one hand, high-dose naringenin can significantly reduce blood glucose levels, and its hypoglycemic effect is comparable to that of insulin (73). On the other hand, a study by Xulu demonstrated that naringin (50 mg/kg) exerts no significant hypoglycemic effect on streptozotocin (STZ)-induced type 1 diabetic rats, but only improves their dyslipidemia and atherosclerosis index (74). Despite the aforementioned controversies, the therapeutic value of naringenin in DKD has been validated by multiple experiments. Specifically, it can effectively reduce proteinuria levels and alleviate the degree of renal fibrosis in model animals. Naringenin can directly up-regulate PPARα/PPARγ protein expression through activation of the CYP4A-20-HETE pathway, and at the same time inhibit the activity of pro-fibrotic factors, such as transforming growth factor-β (TGF-β), and interleukin-1 (IL-1), and ultimately improve the state of lipid metabolism disorders (75).

*Oxybaphus himalaicus Edgew* is a traditional Tibetan medicine approved by the Chinese government. There are still a few studies on modern pharmacology, and the main components and mechanism of action are not completely clear (76). Existing studies have shown that the ethanol extract of this plant can improve DKD by regulating the PPAR signaling pathway (77). Notably, Boeravinone C, a novel natural monomer isolated from *O. himalaicus*, showed bidirectional regulation in DKD mice models and human renal tubular epithelial cells (HK-2) experiments: On the one hand, it activates PPARα transcription factor, enhances FAO ability, and reduces renal tubular lipid accumulation; on the other hand, it inhibits the NF-κB inflammatory pathway and reduces the inflammatory response. The above synergistic effect finally reduces the apoptosis and fibrosis process of renal tubular cells and improves the pathological damage of DKD (78).

Polyphenols can regulate the FAO process of renal tubular cells in DKD by activating PPARα/γ nuclear receptors; at the same time, they can reduce oxidative stress injury and inhibit inflammatory response, thereby delaying the process of renal fibrosis.

### 2.2 Phenolic acids

Phenolic acids are a type of natural phenolic compounds found in various plant-based foods, existing in forms such as amides, esters, or glycosides, and possessing multiple biological activities. Experiments have shown that phenolic acids can regulate lipid metabolism in liver cells by modulating the AMPK, SREBP1, and ACC signaling pathways, and exert anti-inflammatory effects by regulating the TLR, NF-κB, and NLRP3 pathways (79). Epidemiological studies show that phenolic acids have properties including but not limited to anticancer, cardioprotective, anti-inflammatory, immunomodulatory, and anti-obesity effects (80). Existing studies have shown that phenolic acids can improve renal fibrosis in the DKD model mice and regulate autophagy, oxidative stress, and related lipid metabolism disorders, particularly through the FAO process.

Protocatechuic acid (PCA), also known as 3,4-dihydroxybenzoic acid, is a phenolic acid compound that naturally exists in a variety of vegetables and fruits. It is also the main active ingredient of traditional Chinese medicine, such as *Salvia miltiorrhiza* and Hibiscus (81). Mechanism studies have shown that PCA improves metabolic disorders by activating the AMPK/SIRT1 signaling pathway, enhancing mitochondrial biogenesis, and inhibiting NF-κB-mediated inflammatory response (82). Its renal protective effect has been confirmed in a variety of experimental models, which can effectively exert multiple effects such as anti-inflammatory, anti-oxidation, and anti-bacterial (81). Pharmacokinetic studies show that PCA has difficulty penetrating the skin barrier and the blood–brain barrier, with a moderate plasma protein binding rate, but good metabolic stability (83). In the rat model of kidney injury, the absorption of PCA was accelerated and its elimination was slowed, indicating that renal function impairment altered its pharmacokinetic characteristics (84). Existing research has not involved human clinical trials related to the treatment of kidneys with protocatechuic acid, focusing only on rat models. It was found that PCA could restore the mRNA expression levels of PPARα and PPARγ in renal tissue, reduce the concentrations of advanced glycation end product receptor (RAGE), advanced glycation end product (AGEs) and glycated albumin, and promote the reconstruction of PPARγ expression in diabetic mice, improve renal metabolic homeostasis and reduce DKD damage (85). Furthermore, multiple studies have demonstrated that PCA can increase the level of FAO in metabolic tissues: specifically, dietary supplementation with PCA enhances the protein abundance and enzymatic activity of CPT1 in the liver and adipose tissue, and relevant mechanistic studies have been conducted in hepatic cell models (86, 87). However, to date, no studies have directly evaluated whether PCA can restore or enhance FAO function in renal tubular cells or DKD models. Given the core regulatory role of renal tubular FAO in the pathophysiological progression of DKD, in-depth exploration of the tissue-specific regulatory effect of PCA on FAO in renal tissues holds significant research value and feasibility, with potential prospects for clinical translation. Finally, although PCA is considered to be a relatively safe natural phenolic acid PCA is considered a relatively safe natural phenolic acid, systematic toxicological studies, long-term safety evaluations, and research on drug metabolism interactions (CYP/UGT) are still required before its application in DKD.

### 2.3 Alkaloid

Alkaloids are secondary metabolites widely found in plants, characterized by unique cyclic structures containing nitrogen atoms and extensive biological activity, playing an indispensable role in the human body and the field of medicine. Under alkaline conditions, alkaloids can exhibit lipid membrane permeability and are often used as various clinical drug formulations to treat diseases such as cancer, rheumatoid arthritis, and cardiovascular issues (88). Research has found that alkaloids such as sanguinarine can affect cell proliferation and invasion activity through the JAK/STAT signaling pathway (89). Papaverine can upregulate the levels of cAMP and cGMP in cells, relaxing vascular smooth muscle (90). Eriocitrin can influence platelet activation by regulating the PI3K/Akt/GSK3β signaling pathway (91). In recent years, research has found that berberine, as an alkaloid, can play a role in the treatment of DKD by affecting lipid metabolism.

Berberine (BBR), also known as berberine, is a quaternary ammonium alkaloid isolated from the traditional Chinese medicine Rhizoma Coptidis, which has a long history of conventional medicine. BBR has been used in the therapeutic intervention of DKD due to its multiple pharmacological effects, including blood glucose lowering, anti-inflammatory, anti-fibrotic, and gut microbiota regulation (92, 93). Studies have confirmed that BBR can improve lipid metabolism disorders by regulating PPARγ, thereby reducing insulin resistance (94). Research shows that berberine has low bioavailability and potential toxicity in pharmacology, and structural modifications are needed to improve its pharmacokinetic properties (95). A phase 4 clinical trial (NCT01697735) indicated that berberine can improve metabolic diseases such as type 2 diabetes and hyperlipidemia. A clinical trial involving 97 subjects showed that the hypoglycemic effect of berberine was similar to that of metformin (96). The existing meta-analysis also confirmed that berberine has a hypoglycemic effect on type 2 diabetic patients with different levels of fasting plasma glucose (FPG) and glycosylated hemoglobin (HbA1c) (97). In recent years, mechanism studies have found that BBR promotes -FAO through dual pathways: On the one hand, by up-regulating the expression of PPARα/CPT1A/ACOX1 pathway, the FAO capacity of renal tubular epithelial cells was enhanced and lipid accumulation was reduced; on the other hand, the AMPK/PGC-1α axis is activated to repair mitochondrial function, thereby promoting PPARα activity. The above synergistic effect further strengthens the FAO process and ultimately alleviates the pathological process of DKD renal tubular injury and fibrosis (98). BBR improves DKD renal tubular lesions by targeting mitochondrial function reconstruction and fibrosis inhibition. A large body of existing *in vivo* and *in vitro* studies has confirmed that BBR exerts its effects by promoting the expression related to FAO. However, these research conclusions are mostly based on cell experiments or rodent models, and further exploration and verification are still needed for the evidence supporting the effectiveness of its clinical translation.

Coptisine is a protoberberine isoquinoline alkaloid primarily derived from Coptis chinensis Franch., a traditional Chinese herbal medicine. It exhibits diverse pharmacological activities, including anti-inflammatory effects, metabolic regulation, and anti-cancer properties (99). Pharmacokinetic studies have shown that the oral bioavailability of coptisine in mice is extremely low, approximately 8%, primarily attributed to limited intestinal absorption and extensive first-pass hepatic metabolism. This unfavorable pharmacokinetic property may restrict its systemic exposure and therapeutic efficacy (100). To date, no human clinical trials have evaluated coptisine; existing evidence derives from *in vitro*, animal, and pharmacokinetic studies. Coptisine has demonstrated renoprotective effects in experimental models of DKD, including attenuation of oxidative stress and inflammation in streptozotocin (STZ)-induced diabetic rats (101, 102). Recent studies have demonstrated that in renal tubular epithelial cells (HK-2), coptisine can activate AMPK, enhance the phosphorylation of ACC, and upregulate the expression of CPT-1, thereby reducing lipid accumulation under high glucose and palmitic acid conditions (103). These findings suggest that coptisine may affect FAO-related pathways and exert a renoprotective effect in DKD.

### 2.4 Glycoside

Glycosides are natural compounds formed by the connection of lipophilic aglycones and hydrophilic sugar moieties through glycosidic bonds, widely distributed in plants. Glycosides can exert therapeutic effects by regulating key physiological processes such as inflammation, oxidative stress, apoptosis, and fibrosis through pathways like PPARγ/AKT/AMPK and NLRP3/NF-κB (104). Clinically, cardiac glycosides such as digoxin, oleandrin C, and bufalin K have been widely used in the treatment of cardiovascular diseases (105). Recent studies have found that glycosides can play a role in the treatment of DKD by improving podocyte injury, reducing proteinuria, and regulating lipid metabolism.

Glycyrrhizic acid (GA) is a triterpenoid saponin extracted from the roots of Glycyrrhiza uralensis Fisch. It not only has a long history of medicinal use but also has been proven to exert biological effects in multiple fields, including anti-inflammation, antioxidant activity, and anti-diabetic effects (106). Defects in FAO within renal tubular epithelial cells represent a major driving factor underlying lipid accumulation, lipotoxicity, and interstitial fibrosis in chronic kidney disease (CKD) and DKD (107). In recent years, experiments have reported the renal protective effect of GA in DKD. In the db/db mouse model, GA treatment activated the AMPK/SIRT1/PGC-1α signaling pathway, which was accompanied by a reduction in proteinuria and improvement in renal histopathology (108). The activation of AMPK, SIRT1, and PGC-1α has been shown to restore mitochondrial biogenesis and enhance the fatty acid oxidation (FAO) process. This protective effect has been validated in animal experiments and human clinical studies, which can protect the kidney from lipotoxic damage (45, 109). Despite the lack of direct evidence that GA upregulates FAO-related enzymes in the kidney, studies using a hepatocellular model of non-alcoholic fatty liver disease (NAFLD) have shown that GA increases the expression of FAO-related genes, such as PPARα and CPT1A, thereby reducing hepatic steatosis (110). Collectively, these findings suggest that GA may alleviate DKD at least in part by activating the AMPK/SIRT1/PGC-1α axis, which is closely associated with mitochondrial energy metabolism and FAO. However, further mechanistic studies are required to determine whether the renal protective effect of GA in DKD is directly mediated by the restoration of renal FAO.

In summary, glycoside compounds can promote mitochondrial biosynthesis by activating the AMPK/PPARγ/PGC-1α axis, and double block inflammation and fibrosis pathways, significantly reducing early diabetic kidney injury in db/db mice.

## 3 Discussion

Although significant progress has been made in related research, the specific molecular mechanism of natural compounds intervention in DKD still needs to be further explored. Currently, clinical treatment mainly relies on symptomatic therapy such as glycemic control, blood pressure control, and lipid reduction. However, there is an absence of drugs specifically targeting the pathophysiology of DKD. Moreover, conventional medications have limited effects in delaying or controlling the progression of DKD, cannot completely block disease deterioration, and may also cause hyperkalemia and fluctuations in renal function. The association between FAO and DKD is not yet fully understood, and the relationship with mitochondrial autophagy still needs to be clarified. Existing research lacks studies on the interaction between FAO and key pathways of DKD, and the relevant signaling networks have not been clarified. In clinical practice, FAO modulators have not yet been involved in the research of DKD. Furthermore, fatty acid metabolism is closely related to glucose and lipid disorders, but regulating FAO may lead to metabolic imbalances, necessitating a strict assessment of safety. The chemical composition of natural compounds is inherently complex and unstable, which significantly impacts the reliability and reproducibility of research findings. Most bioactive components derived from natural compounds still lack systematic and comprehensive pharmacokinetic and pharmacodynamic research data, and they generally suffer from the issue of relatively low bioavailability. The pharmacological mechanism is not yet fully understood, and some practical components need further exploration and validation; moreover, the potential renal toxicity risks of these components have not been systematically studied. A large number of mechanistic studies are based on cell or animal models, which have limitations in simulating the pathology of late-stage DKD in humans, and there is a lack of high-quality human clinical trials. DKD is often induced by diabetes and is prone to multiple complications. Future research needs to closely combine pharmacological effects with toxicological assessment, attach great importance to safety in clinical exploration, and examine its potential for multi-target comprehensive regulation.

## 4 Future prospectus

The aforementioned challenges also highlight new directions for future research, with the goal of advancing the clinical translation of natural compounds targeting FAO in DKD. First, from the perspective of clinical feasibility, it is imperative to establish rigorous and standardized production processes and quality control systems to ensure the stability and reproducibility of bioactive components. Concurrently, the pharmacokinetic (ADME) and pharmacodynamic profiles of these compounds in healthy individuals and patients with DKD at different stages should be fully elucidated. Emerging technologies are expected to enhance bioavailability, improve tissue specificity, and reduce potential toxicity, thereby supporting early-phase clinical trials. Validating molecular targets, signaling pathways, and interaction networks using organoid and disease-mimicking models will lay a solid foundation for subsequent Phase I/II clinical trials, while robust Phase III randomized controlled trials (RCTs) are needed to further clarify efficacy and safety based on these preliminary findings.

Second, systematic, biomarker-based strategies should be employed to link molecular mechanisms with clinical outcomes. Lipidomic profiles and renal FAO enzyme expression levels can serve as pharmacodynamic indicators to evaluate therapeutic efficacy and guide patient stratification. Priority should be given to natural compounds with well-defined FAO-modulating effects for conducting high-quality RCTs, incorporating clinically relevant endpoints and patient-reported outcomes (PROs). Additionally, the combined use of natural compounds with drugs proven to have renoprotective effects (e.g., SGLT2 inhibitors, GLP-1 receptor agonists, or RAAS blockers) may exert synergistic effects in improving lipid metabolism and delaying DKD progression.

Finally, existing evidence demonstrates that enhancing local renal FAO can effectively reduce lipid accumulation, improve mitochondrial function, and alleviate fibrosis (111). However, there is no reliable evidence to support the claim that “upregulating renal FAO alone can restore systemic lipid levels to a healthy state.” In contrast, clinical data indicate that enhanced systemic FAO (particularly in the liver) can significantly improve lipid profiles (112, 113). Therefore, the value of FAO upregulation in DKD primarily lies in its local renoprotective effects, while its role in the systemic circulation remains to be further elucidated. In summary, FAO modulation represents a promising emerging direction for natural compounds-based DKD therapy, but comprehensive validation of its safety, efficacy, and clinical application value through systematic translational research is still required.

## 5 Conclusion

Our review found that natural compounds targeting FAO-related signaling pathways hold promise as important adjuvant therapeutic agents in the treatment of DKD. Polyphenols, phenolic acids, alkaloids, and glycosides can all affect the progression of DKD. They mainly improve lipid metabolism and deposition by regulating pathways such as PPARα/γ, AMPK/SIRT1/PGC-1α, and their downstream effector proteins, thereby alleviating podocyte injury and the process of fibrosis. In summary, with its multi-target synergistic regulation characteristics, natural compounds can not only directly improve oxidative stress injury and lipid metabolism disorder of DKD, but also restore renal function by regulating the FAO process, which provides a distinctive and potential intervention strategy for DKD treatment.
